# Platycodin D and voluntary running synergistically ameliorate memory deficits in 5 × FAD mice via mediating neuromodulation and neuroinflammation

**DOI:** 10.3389/fnagi.2024.1451766

**Published:** 2024-09-25

**Authors:** Junxin Liu, Jiahui Jiang, Chuantong He, Longjian Zhou, Yi Zhang, Shuai Zhao, Zhiyou Yang

**Affiliations:** ^1^Guangdong Provincial Key Laboratory of Aquatic Product Processing and Safety, Guangdong Province Engineering Laboratory for Marine Biological Products, Zhanjiang Municipal Key Laboratory of Marine Drugs and Nutrition for Brain Health, College of Food Science and Technology, Guangdong Ocean University, Zhanjiang, China; ^2^Collaborative Innovation Center of Seafood Deep Processing, Dalian Polytechnic University, Dalian, China

**Keywords:** Alzheimer’s disease, voluntary running, platycodin D, neuroinflammation, monoamine neurotransmitter

## Abstract

**Introduction:**

Alzheimer’s disease (AD) is the leading cause of dementia, and currently, no effective treatments are available to reverse or halt its progression in clinical practice. Although a plethora of studies have highlighted the benefits of physical exercise in combating AD, elder individuals often have limited exercise capacity. Therefore, mild physical exercise and nutritional interventions represent potential strategies for preventing and mitigating neurodegenerative diseases. Our research, along with other studies, have demonstrated that platycodin D (PD) or its metabolite, platycodigenin, derived from the medicinal plant *Platycodon grandiflorus*, exerts neuroprotective effects against amyloid β (Aβ)-induced neuroinflammation. However, the combined effects of PD and physical exercise on alleviating AD have yet to be explored. The current study aimed to investigate whether combined therapy could synergistically ameliorate memory deficits and AD pathology in 5 × FAD mice.

**Methods:**

Five-month-old 5 × FAD mice were randomly assigned to four groups, and received either PD (5 mg/kg/day, p.o.), voluntary running, or a combination of both for 47 days. Nest building test, locomotion test, and Morris water maze test were used to evaluate the cognitive function. Immunohistochemical and ELISA analysis was performed to determine Aβ build-up, microglia and astrocytes hyperactivation, and survival neurons in the hippocampus and perirhinal cortex. Real-time quantitative PCR analysis was used to assess the polarization of microglia and astrocytes. HPLC analysis was performed to measure monoamine neurotransmitters in the hippocampus.

**Results and discussion:**

The combination of PD and voluntary running synergistically restored nest-building behavior, alleviated recognition and spatial memory deficits, and showed superior effects compared to monotherapy. In addition, the PD and voluntary running combination reduced Aβ build-up, decreased hyperactivation of microglia and astrocytes in the hippocampus and perirhinal cortex, promoted the polarization of inflammatory M1 microglia and reactive astrocytes toward beneficial phenotypes, and lowered systemic circulating pro-inflammatory cytokines while increasing anti-inflammatory cytokines in 5 × FAD mice. Furthermore, combined therapy effectively protected neurons and increased levels of 5-hydroxytryptamine (5-HT) and dopamine (DA) in the hippocampus of 5 × FAD mice. In conclusion, the combination of PD and voluntary running holds great potential as a treatment for AD, offering promise for delaying onset or progression of AD.

## 1 Introduction

Alzheimer’s disease (AD) is a primary neurodegenerative disorder predominantly affecting the elderly. It is characterized by persistent disturbances in higher neurological activities, including consciousness, emotion, memory, analytical judgment, thinking, and spatial recognition (McKhann et al., 2011). A key pathological hallmark of AD is the accumulation of beta-amyloid (Aβ) plaques. Aβ contributes to neurotoxicity by inducing neuroinflammation, promoting hyperphosphorylation of tau proteins, and sustaining neuronal hyperexcitability (Maestú et al., 2021).

Neuroinflammation, a component of the innate inflammatory response of the central nervous system (CNS), initially benefits to eliminate pathogens and maintain brain homeostasis (Gorji, 2022). However, during the pathological progression of AD, neuroinflammation becomes hyperactivated, leading to Aβ accumulation, neuronal damage, and ultimately cognitive deficits (Li et al., 2023). M1 microglia and reactive neurotoxic astrocytes exhibit a pro-inflammatory phenotype, releasing a variety of pro-inflammatory cytokines and accelerating neuronal death, whereas M2 microglia and S100a10^+^ astrocytes are neuroprotective, producing neurotrophic factors and anti-inflammatory cytokines (Liddelow et al., 2017; Wei and Li, 2022). Therefore, prompting astrocytes and microglia to adopt a beneficial polarization is a crucial strategy for AD treatment.

Aβ fibrils and hyperphosphorylated tau proteins can act as toxins, disrupting synaptic plasticity and neurotransmitter release, which leads to learning and cognitive dysfunction (Guerrero-Muñoz et al., 2015). Reduced levels of monoamine neurotransmitters such as dopamine (DA), norepinephrine (NE), and serotonin (5-HT) have been observed in the brains of various AD models and are associated with impaired cognitive memory (Chalermpalanupap et al., 2013; Rodríguez et al., 2012). Thus, modulating monoamine neurotransmitter levels through pharmacological interventions has shown efficacy in restoring cognitive memory capacity (Castellano et al., 2016). Several anti-AD drugs targeting neurotransmitters are either completed or in phase III clinical trials, including escitalopram, brexpiprazole, AVP-786, and nabilone (Cummings et al., 2023). However, there remains a significant gap between clinical trials and widespread clinical applications.

*Platycodon grandiflorum* has been used as a medicine food homology plant in China for centuries, and *Platycodon grandiflorum* pickle is a specialty in Korea. Our previous studies have proved that platycodigenin is effective in ameliorating LPS-induced inflammation and Aβ-induced axonal atrophy and neuronal death (Yang et al., 2019b). Platycodin D (PD) has been shown to improve learning and memory by enhancing neurite outgrowth and synaptogenesis in the mouse hippocampus, as well as ameliorating memory deficits by regulating PI3K/Akt/GSK3β signaling in type 2 diabetes mellitus mice (Kim et al., 2017; Lu et al., 2024). In addition to nutritional interventions, physical exercise is a crucial way to improve cognitive ability in AD. Prolonged sedentary behavior contributes to cognitive decline in the elderly (Edwards and Loprinzi, 2017), whereas planned or voluntary exercise has been shown to enhance cognitive performance in both mice and humans with AD (Kemoun et al., 2010; Mehla et al., 2022). Exercise can also significantly reduce medication-induced side effects in AD treatment. Thus, combining dietary supplement with regular physical exercise may be a more effective strategy for delaying AD progression. We hypothesize that the combination of PD with voluntary exercise could synergistically ameliorate cognitive deficits in 5 × FAD mice more effectively than monotherapy. Hence, the present study investigates the combined effects of these strategies on cognitive abilities and AD-related pathologies in 5 × FAD mice, and examines the underlying molecular mechanisms by assessing monoamine neurotransmitters, inflammatory cytokines, and the polarization of microglia and astrocytes.

## 2 Materials and methods

### 2.1 Animals experiment design

The experimental 5 × FAD mice were purchased from Aniphe BioLab (Jiangsu, China). These mice were maintained as hemizygotes by crossing 5 × FAD males with wild-type C57BL/6 F1 females. All care and experimental protocols were performed in line with the guidelines of the Animal Experimentation Committee of Guangdong Ocean University (SYXK2022-0032). Five FAD mice (5 months old, male) were utilized as treated groups and littermate wild-type C57BL/6 mice (5 months old, male, *n* = 6) were used as control (CT) group. The 5 × FAD mice were randomly assigned to 4 groups: a sedentary group (TgS, *n* = 5), a voluntary running group (TgR, *n* = 5), a PD treatment group (TgS-PD, *n* = 5), and a combined voluntary running and PD treatment group (TgR-PD, *n* = 6). Mice in the voluntary running group were housed in a multichannel animal wheel running system (KEWBASIS, Nanjing, China), and the running distances were recorded daily. To minimize the impact of a single housing on social behavior, each mouse from each group was single caged in standard polypropylene cages throughout the experiment. The temperature was maintained at 23 ± 2° with a 12-h light/dark cycle, and the humanity was kept at 55 ± 10%, all mice had *ad libitum* access to food and water. PD was purchased from PUSH Bio-technology (Chengdu, China) with a purity exceeding 98.5% as confirmed by HPLC analysis. PD was dissolved in d-H_2_O to a concentration of 0.5 mg/ml and intragastrical administered continuously for 47 days at a dose of 5 mg/kg/day. The dosage was determined based on our preliminary experimental results.

### 2.2 Nest building test

Each animal was provided with the same type of nesting material (16 pieces of cotton paper, each measuring 3 cm × 3 cm). These pieces were evenly distributed in the cage to prevent them from sticking together. The nesting patterns were assessed 24 h later and scored based on the following criteria: score 1–little to no nesting material was moved, score 2–nesting material was moved but did not form a distinct nest, score 3–nesting material was moved and aggregated into a flat nest, score 4–nesting material was aggregated into a distinct nest with walls above the mice, score 5–based on score 4, the nesting material was gnawed and shaped into a cozy nest (Deacon, 2006).

### 2.3 Open field test

The open field test was performed to assess the animals’ exploratory behavior, mobility, and anxiety in a novel environment (Kraeuter et al., 2019). Prior to the experiment, the mice were acclimated to a quiet room for 1 h. The experimental chamber was a square box with 40 cm in length, width and height, made of black polyvinyl chloride. The mice were placed head-down in the center of the chamber, and the traveling paths were tracked for 5 min using a digital camera. The moved distance, rearing numbers, and number of feces were recorded with VisuTrack software (Xinruan, Shanghai, China). To prevent odor interference, the chamber was cleaned with 5% ethanol before each animal’s trial.

### 2.4 Novel object recognition test

To assess the learning and memory abilities of mice, a novel object recognition test was performed based on their innate exploring tendency to novel objects (Schlunk et al., 2021). The experimental protocol was adapted with minor modifications from previous studies (Deng et al., 2022). During the training phase, two identical objects were placed symmetrically on opposite diagonals of the chamber. The mice were introduced into the chamber, facing the wall at the corners equidistant from the two objects, and the exploratory times with each object was recorded within 8 min. One hour later, one of the objects was replaced with a novel object, and the preferential index for the novel object was calculated.

### 2.5 Morris water maze test

The Morris water maze (MWM) test is commonly used to assess the spatial memory abilities of mice (Othman et al., 2022). A circular pool, 50 cm high and 120 cm in diameter, equipped with a video-tracking system (Shanghai Xinruan Information Tech, Shanghai, China) was employed. The pool was filled with water to a depth of 30 cm, mixed with titanium dioxide, and maintained at a temperature of 22 ± 1°C. Four equidistant points N, E, S, W were marked with brightly colored shapes on the wall of the pool as signposts, dividing the pool into four quadrants: NW, WS, SE, and EN. A transparent circular platform, 29 cm high and 12 cm in diameter, was submerged 1 cm below the water surface in one of the quadrants. Mice were sequentially placed in each of the four quadrants daily and allowed 1 min to locate the hidden platform. Escape latency, defined as the time taken for the mice to find the platform (3 s retention), was recorded. The mice that did not find the platform within 60 s were manually guided to it and allowed to acclimate for 15 s. Four trials per day were conducted in each different quadrant with 20 min intervals between trials over 5 consecutive days. On day 6, the platform was removed, and the mice were placed in the quadrant that is opposite to the platform. A probe trial was conducted for 1 min, during which the number of platform crossings and the time spent in the target quadrant were recorded.

### 2.6 Mouse tissue preparation

Voluntary running was ceased for 1 day prior to the sacrifice of the mice to minimize acute stress. The mice were deeply anesthetized with a mixture of xylazine hydrochloride (23 mg/kg), zolazepam (15 mg/kg), and salbutamol hydrochloride (15 mg/kg) administered via intraperitoneally injection. Approximately 1.5 ml of blood was collected from the abdominal aorta, centrifuged at 2,500 *g* for 15 min, and the serum was harvested. Cardiac perfusion was performed to remove the circulating blood. The left hemisphere of the brain was immediately soaked in 4% paraformaldehyde (Biosharp, Guangzhou, China) and stored at 4°C for fixation. The hippocampus and prefrontal cortices were dissected from the right hemisphere, rapidly frozen in liquid nitrogen, and stored at −80°C.

### 2.7 Enzyme linked immunosorbent assay (ELISA) analysis

Thirty milligrams of cortex tissue was lysed on ice for 30 min using Mammalian Protein Extraction Reagent (M-PER™, Thermo Scientific, Massachusetts, USA) containing 1× protease inhibitor mix. The lysate was then centrifuged at 12,000 *g* at 4°C for 10 min. The supernatant was collected, and total protein concentration was measured using the Pierce™ 660 nm Protein Assay Kit (Thermo Scientific, Waltham, Massachusetts, USA). Levels of Aβ1-42 in the cortex and concentrations of IL-1β, TNF-α, IL-4, and IL-10 in serum were quantified using ELISA kits (Zeyu Biological, Jiangsu, China).

### 2.8 Immunohistochemistry analysis

The left hemisphere of the brain was picked from paraformaldehyde post 2 days of fixation. The surface liquid was removed with filter paper, and the tissue was then dehydrated through a gradient of 10, 20, and 30% sucrose solutions. The samples were embedded in Sakura Tissue-Tek^®^ O.C.T. Compound, and 15 μm thick cryosections were made by a cryostat (Kedee, Jinhua, China). Aβ (1:500, 700254, Invitrogen), ionized calcium-binding adaptor molecule 1 (Iba1, 1:500, 019-19741, Wako), glial fibrillary acidic protein (GFAP) (1:200, MA5-12023, Invitrogen), NeuN (1:500, ab177487, Abcam) were used as primary antibodies, and goat anti-mouse (Alexa Fluor 594, 1:500, ab150116) and goat anti-rabbit (Alexa Fluor 488, 1:500, ab150081) IgG were used as secondary antibodies. Counterstaining was performed using DAPI (1 μg/ml, MCE, USA). The immunopanned slices were captured using an ECHO Revlove fluorescence microscope (ECHO, San Diego, California, USA) and quantitatively analyzed with ImageJ software (NIH, Bethesda, Maryland, USA).

### 2.9 High performance liquid chromatography (HPLC) analysis

Approximately 15 mg of hippocampal tissue was mixed with 150 μL of lysis buffer (0.6 mol/L perchloric acid, 0.5 mmol/L disodium ethylenediaminetetraacetic acid, and 0.1 g/L L-cysteine), homogenized, and centrifuged twice for 15 min each at 14,000 *g* and 4°C. The collected supernatant was then mixed with an equal volume of perchloric acid precipitant (1.2 mol/L dipotassium hydrogen phosphate, 2 mmol/L disodium ethylenediaminetetraacetic acid) and allowed to stand for 10 min in an ice bath. The mixture was centrifuged for 15 min at 14,000 *g* and 4°C, and the resulting supernatant was filtered through a 0.45 μm membrane. A 20 μL aliquot of the filtrate was injected into an Agilent 1260 Infinity II system (Agilent, Santa Clara, California, USA) equipped with an Agilent ZORBAX 300SB-C18 column (150 mm × 4.6 mm, 5 μm, Agilent, Santa Clara, California, USA) and an Agilent 1260 infinity fluorescence detector (excitation wavelength at 280 nm and emission wavelength and 330 nm). The mobile phase consisted of citrate sodium acetate buffer (A) containing 0.5 mM C_7_H_15_O_3_SNa, 0.5 mM Na_2_-EDTA, and 5 mM C_6_H_15_N, and methanol (B) at an isocratic elution with 87% A. The flow rate was set at 1.0 mL/min. The contents of dopamine (DA), dihydroxyphenylacetic acid (DOPAC), 5-hydroxytryptamine (5-HT), 5-hydroxyindoleacetic acid (5-HIAA), norepinephrine (NE), and 3-methoxy-4-hydroxyphenylglycol (MHPG) were quantified according to standard curve (Yang et al., 2021).

### 2.10 Real-time quantitative PCR analysis

Total RNA was extracted from 10 mg of mouse hippocampal tissue using AG RNAex Pro Reagent (Accurate Biology, Hunan, China). The tissue was thoroughly ground, left to stand for 15 min at room temperature, then mixed with 200 μL of chloroform, vortexed for 30 s, and centrifuged at 12,000 *g* for 15 min at 4°C post an additional 15-min standing period. The supernatant was collected, mixed with an equal volume of isopropanol, left to stand for 10 min, and then centrifuged at 12,000 *g* for 10 min at 4°. The RNA pellets were washed twice with 75% ethanol, air-dried, and then resuspended in 15–20 μL of DEPC water. RNA concentration was measured using a DS-11 Ultramicro spectrophotometer (DeNovix, USA). cDNA was synthesized from RNA using the HiScript II Q Select RT SuperMix for qPCR (+gDNA wiper) (Vazyme, China) kit. PCR amplification was performed with ChamQ Universal SYBR qPCR Master Mix Kit, and mRNA expression levels were measured using a CFX96Touch TM Real-Time Fluorescent Quantitative PCR System (Bio-Rad, Hercules, California, USA) with initial activation at 95°C for 30 s, subsequently by 40 cycles of amplification (5 s at 95°C and 30 s at 60°C). Primer sequences for mouse CD11b, CD206, C3, S100a10, and actin (Sango Biotech, Shanghai, China) were as follows: CD11b forward: TATGGAGCATCAATAGCCAGCCT, CD11b reverse: GAGAT CCTTACCCCCACTCAGAGAC; CD206 forward: TCTTTGCC TTTCCCAGTCTCC, CD206 reverse: TGACACCCAGCGG AATTTC, C3 forward: AGCTTCAGGGTCCCAGCTAC, C3 reverse: GCTGGAATCTTGATGGAGACGC; S100a10 forward: GTTTGCAGGCGACAAAGACC, S100a10 reverse: ATTTTGTCCACAGCCAGAGG; Actin forward: CATCCGTAAAGACCTCTATGCCAAC, Actin reverse: ATGGAGCCACCGATCCACA.

### 2.11 Statistical analysis

Results from behavioral and molecular experiments were processed using GraphPad Prism 9 (GraphPad Software, California, USA) or “rcompanion” package (for the Scheirer-Ray-Hare test) of R version 4.2.3. Data are presented as mean ± SEM. For multigroup comparisons involving data characterized by non-normal distribution, heteroscedasticity, or small sample sizes, the Kruskal–Wallis test followed by Dunn’s *post-hoc* test was employed for one-factor designs. In the case of two-way designs, the Scheirer-Ray-Hare test, an extension of the Kruskal–Wallis test, was utilized, followed by Dunn’s *post-hoc* test. The *p* < 0.05 was indicated as statistical significance.

## 3 Results

### 3.1 Voluntary exercise and PD synergically ameliorated memory deficits in 5 × FAD mice

**FIGURE 1:**
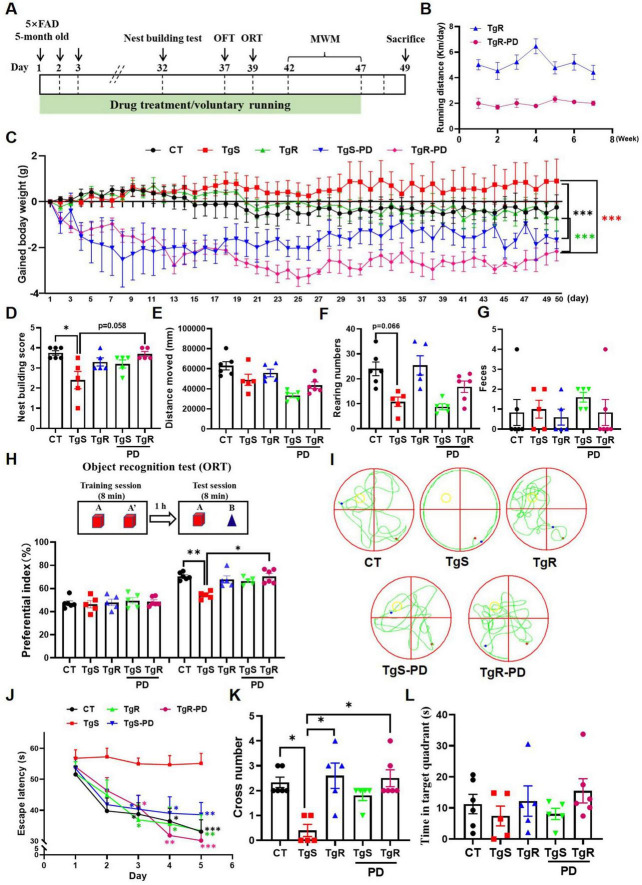
PD and voluntary running ameliorated cognitive deficits in 5 × FAD mice. **(A)** The experimental schedule. **(B)** Running distance of TgR and TgR-PD mice. **(C)** The gained body weight. **(D)** Score in the nest building test. **(E)** Total distance moved in the open field test. **(F)** Rearing numbers in the open field test. **(G)** The number of feces in the open field test. **(H)** Novel object recognition (NOR) memory test. **(I)** Representative swimming paths on the 6th day of the MWM. **(J)** Escape latency in the MWM. **(K)** Platform crossing numbers on the 6th day in the probe trial test. **(L)** Time spent in target quartant on the 6th day in the probe trial test. *p < 0.05, **p < 0.01, ***p < 0.001 vs. TgS group. The Kruskal–Wallis test followed by Dunn’s *post-hoc* test was used for one-factor designs, and the Scheirer-Ray-Hare test (an extension of the Kruskal–Wallis test) followed by Dunn’s *post-hoc* test was used for experiments with two-way designs (mean ± SEM, *n* = 5–6).

To determine the optimal dosage of PD, we conducted a preliminary experiment. PD was administered orally to 5 × FAD mice (6–8 months old, half male and female) at doses of 5 and 15 mg/kg for 20 days. Behavioral tests, including the locomotion test, object recognition test (ORT), elevated maze test, and object location test (OLT) were performed on days 14, 15, 18, and 19, respectively (Supplementary Figure 1A). Compared to vehicle-treated 5 × FAD mice, PD-treated mice showed a trend toward decreased body weight (Supplementary Figure 1B). The 5 × FAD mice displayed significantly reduced spontaneous activity compared to wild-type mice (Supplementary Figure 1C), while vehicle-treated 5 × FAD mice spent more time in the central zone compared to wild-type and PD-treated mice (Supplementary Figure 1D), indicating PD improved exploratory behavior. No significant differences were observed in the ratio of open and closed arm entries between groups (Supplementary Figure 1E). Vehicle-treated 5 × FAD mice failed to recognize novel or novel-placed objects compared to wild-type mice, whereas PD-treated mice showed significantly increased preferential index for novel object or novel-placed object (Supplementary Figures 1F, G). We also evaluated the impact of voluntary running on memory function in 5 × FAD mice. Experiments were performed using 5 × FAD mice (2.5–3 months old, half male and female, *n* = 8) and littermate wild-type C57BL/6 mice (2.5–3 months old, half male and female, *n* = 8). They were randomly assigned to 4 groups: sedentary 5 × FAD, sedentary wild-type, voluntary running 5 × FAD, and voluntary running wild-type. Even a month of voluntary running significantly improved spatial memory in 5 × FAD mice (Supplementary Figure 2A–G). To further assess the combined effects of voluntary exercise and PD treatment on memory deficits, we orally administered PD (5 mg/kg/d) with voluntary excise to 5 × FAD mice for 47 consecutive days (Figure 1A). The running distance for the TgR group averaged approximately 5 km per day, while the TgR-PD showed a marked decrease to 2 km per day (Figure 1B). We monitored the changes of body weight during voluntary running and/or PD treatment, the TgR-PD group had significantly reduced body weight compared to the TgS and TgR groups (Figure 1C and Supplementary Figure 3). The TgR or TgS-PD groups also showed decreased body weight compared to the TgS 5 × FAD mice (Figure 1C), indicating that the combination of PD and physical exercise more efficiently reduced body weight than either treatment alone. Nest-building behavior, a natural indicator of overall health and cognitive function in mice, was assessed. Compared to wild-type mice, sedentary 5 × FAD mice exhibited severely impaired nest-building ability. The TgR-PD group demonstrated significantly improved nest-building ability compared to the TgS group, and showed slightly better scores than the TgR and TgS-PD groups (Figure 1D).

The open field test was used to evaluate the mice’s exploratory behavior and mobility. PD administration tended to decrease spontaneous activity in the mice (Figure 1E), which corresponded with the voluntary running distance shown in Figure 1B. The number of rearing was dramatically lower in the TgS group compared to the CT group, while the TgR group exhibited a rebound effect, and the TgR-PD group showed an increased trend compared to the TgS-PD group (Figure 1F), indicating that voluntary running enhanced exploratory behavior. The number of feces did not exhibit any significance among groups (Figure 1G). In the object recognition test, mice were equal to explore the two identical objects during the training session. During the test session, TgS mice failed to recognize the novel objects compared to CT mice (**p < 0.01, TgS vs. CT), whereas the preferential index for novel objects was significantly increased in the TgR-PD group (*p < 0.05, TgS vs. TgR-PD), displaying the best discrimination between old and novel objects (Figure 1H). In the MWM test, the escape latency for the TgS group was significantly higher than that of the other groups, with the TgR-PD group showing the shortest escape latency from day 4 (Figure 1J). To further assess the memory retention following exercise and PD intervention, a spatial probe trial was conducted on day 6. Compared to TgS mice, the number of platform crossings was significantly increased in the TgR-PD, TgR, and TgS-PD groups (Figures 1I, K), with a similar trend observed in the time spent in the target quadrant (Figure 1L). In conclusion, the combination of PD and voluntary exercise synergistically ameliorated cognitive deficits in 5 × FAD mice, demonstrating superior efficacy compared to monotherapy.

### 3.2 PD and voluntary exercise attenuated Aβ plaques build-up in 5 × FAD mice

**FIGURE 2:**
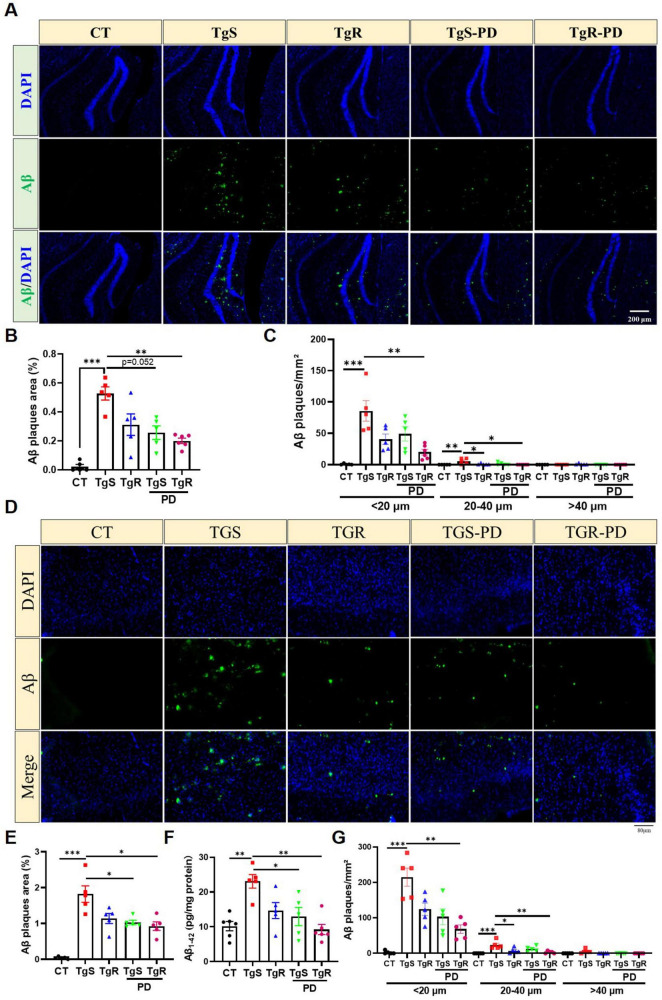
PD and voluntary running reduced Aβ deposition in the brain of 5 × FAD mice. **(A)** Representative Aβ immunopanned photos in the hippocampus. **(B)** The percentage of plaque total area in the hippocampus. **(C)** Number of Aβ1-42 plaques in different sizes (diameter < 20 μm, 20–40 μm, > 40 μm) per mm^2^ in hippocampus. **(D)** Representative Aβ immunopanned photos in perirhinal cortex. **(E)** The percentage of plaque total area in perirhinal cortex. **(F)** Relative expression of Aβ1-42 in perirhinal cortex measured by ELISA analysis. **(G)** Number of Aβ1-42 plaques in different sizes (diameter < 20 μm, 20–40 μm, > 40 μm) per mm^2^ in perirhinal cortex. *p < 0.05, **p < 0.01, ***p < 0.001 vs. TgS group. The Kruskal–Wallis test followed by Dunn’s *post-hoc* test was used (mean ± SEM, *n* = 5–6).

Extracellular Aβ aggregation, which forms senile plaques, is one of the main pathological features of AD. We performed Aβ immunofluorescence staining and ELISA assays in the hippocampus and perirhinal cortex. The analysis revealed a significant increase in plaque sizes (diameter < 20 μm, 20–40 μm, and > 40 μm) and plaque areas in both the hippocampus (Figures 2A–C) and perirhinal cortex compared to CT mice (Figures 2D–G). In 5 × FAD mice, the majority of Aβ plaques were in the < 20 μm diameter range (Figures 2C, G). The TgR-PD group showed a reduction in Aβ plaques compared to the PD or voluntary running alone groups (Figures 2B, E). No plaques were detected in the wild-type CT mice. In addition, an ELISA kit was used to examine Aβ_1–42_ levels in the prefrontal cortex (Figure 2F). Aβ_1–42_ levels were unambiguously upregulated in TgS mice compared to CT mice, but PD treatment or physical running significantly reduced Aβ accumulation. The TgR-PD group exhibited a decreasing trend in Aβ_1–42_ levels compared to the other two single interventions.

### 3.3 PD and voluntary exercise modulated neuroinflammation in 5 × FAD mice

**FIGURE 3:**
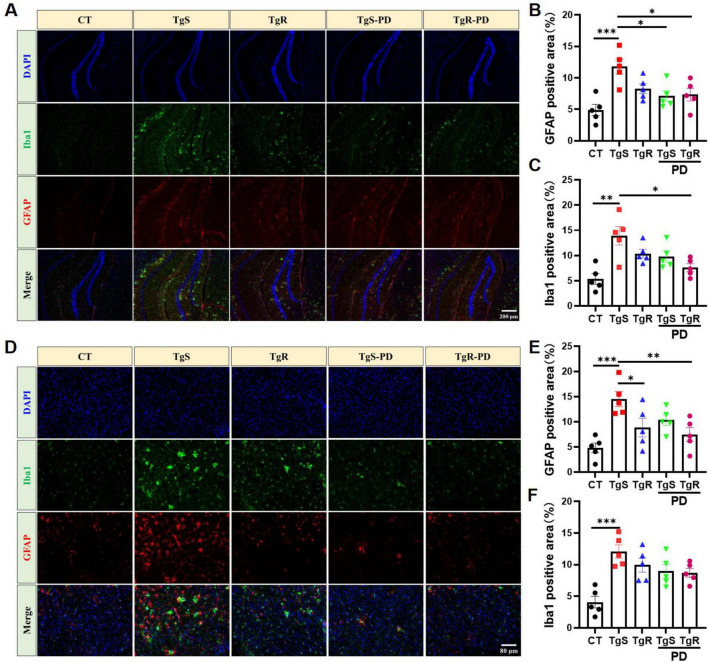
PD and voluntary running suppressed microglia and astrocytes hyperactivation in 5 × FAD mice. **(A)** Representative Iba1 and GFAP immunostaining images in the hippocampus of mice. **(B)** Fluorescence expression of GFAP in the hippocampus. **(C)** Fluorescence expression of Iba1 in the hippocampus. **(D)** Representative Iba1 and GFAP immunostaining images in the perirhinal cortex of mice. **(E)** Fluorescence expression of GFAP in the perirhinal cortex. **(F)** Fluorescence expression of Iba1 in the perirhinal cortex. *p < 0.05, **p < 0.01, ***p < 0.001 vs. TgS group. The Kruskal–Wallis test followed by Dunn’s *post-hoc* test was used (mean ± SEM, *n* = 5).

Hyperactivated microglia and astrocytes, marked by Iba1 and GFAP, respectively, were remarkably upregulated in the hippocampus (Figures 3A–C) and perirhinal cortex (Figure 3D–F) of sedentary 5 × FAD mice compared to CT mice. TgR-PD treatment significantly reduced Iba1 expression in the hippocampus and cortex (Figures 3C, F) and was more effective at suppressing GFAP compared to TgR or TgS-PD alone (Figures 3B, E).

**FIGURE 4:**
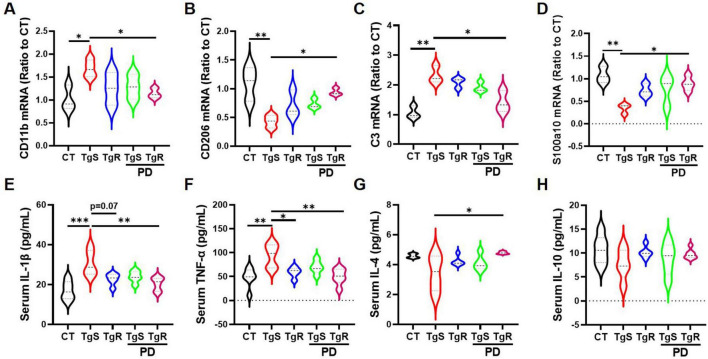
PD and voluntary running promoted the polarization of microglia and astrocytes to beneficial phenotypes in the hippocampus. **(A)** mRNA expression of M1 microglia marker CD11b. **(B)** mRNA expression of M2 microglia marker CD206. **(C)** mRNA expression of A1 astrocyte marker C3. **(D)** mRNA expression of reactive astrocyte marker S100a10. **(E–H)** IL-1β, TNF-α, IL-4, and IL-10 expression level in serum by ELISA analysis. *p < 0.05, **p < 0.01, ***p < 0.001 vs. TgS group. The Kruskal–Wallis test followed by Dunn’s *post-hoc* test was used (mean ± SEM, *n* = 3–4).

To assess microglia and astrocyte polarization toward beneficial phenotypes, we conducted qRT-PCR analysis for markers C3 and S100a10 (reactive astrocytes), and CD11b and CD206 (M1 and M2 microglia, respectively) (Miyamoto et al., 2020; Yang et al., 2019a). CD11b expression was significantly up-regulated in the TgS group compared to the CT group, while it was down-regulated in the TgR-PD group (Figure 4A). CD206 expression was significantly up-regulated in the TgR-PD group and the wild-type CT group compared to sedentary 5 × FAD mice (Figure 4B). C3 mRNA expression was up-regulated while S100a10 was down-regulated in the TgS group compared to CT mice, PD combined with voluntary running effectively restored the imbalance of reactive astrocytes (Figures 4C, D). These findings suggest that PD combined with voluntary running synergistically promotes the polarization of microglia and astrocytes toward an anti-inflammatory phenotype. ELISA analysis of serum inflammatory cytokines revealed significant increases in TNF-α and IL-1β compared to CT mice (Figure 4E–H), which were markedly reversed by TgR-PD and TgR treatments (Figure 4E, F). The level of IL-4 was significantly increased in TgR-PD and TgR groups compared to the TgS group (Figure 4G), while IL-10 levels remained unchanged among groups.

### 3.4 PD and voluntary running modulated neuronal activity in 5 × FAD mice

**FIGURE 5:**
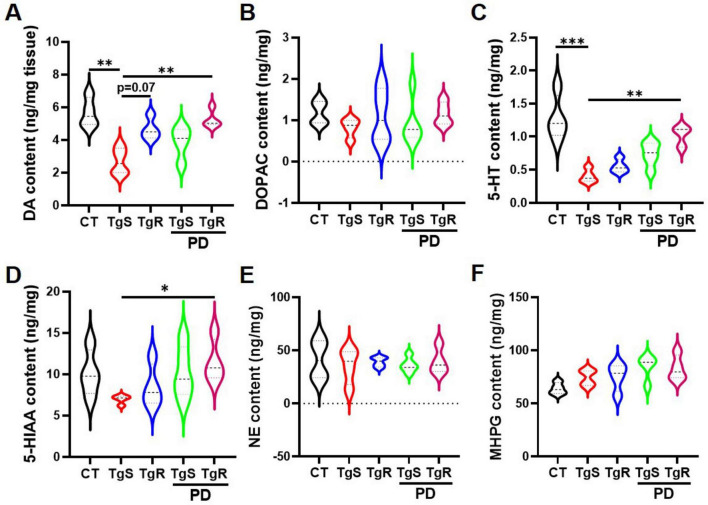
PD and voluntary running modulated monoamine neurotransmitters in the hippocampus. **(A–F)** Contents of DA, DOPAC, 5-HT, 5-HIAA, NE, and MHPG determined by HPLC analysis. *p < 0.05, **p < 0.01, ***p < 0.001 vs. TgS group. The Kruskal–Wallis test followed by Dunn’s *post-hoc* test was used (mean ± SEM, *n* = 4).

The neurotransmitter system is closely linked to learning and memory. To investigate how PD and/or physical running affect neurotransmitter levels, we analyzed the expression of DA, 5-HT, NE, and their metabolites DOPAC, 5-HIAA, and MHPG in the hippocampus via HPLC analysis (Figure 5). Results showed that levels of 5-HT and DA were significantly increased in the TgR-PD group and the CT group compared to the TgS group, with trends toward increased levels also observed in the TgR and TgS-PD groups (Figures 5A, C). However, expression levels of DOPAC, 5-HIAA, NE, and its metabolite MHPG were not dramatically changed among groups (Figures 5B, D–F).

**FIGURE 6:**
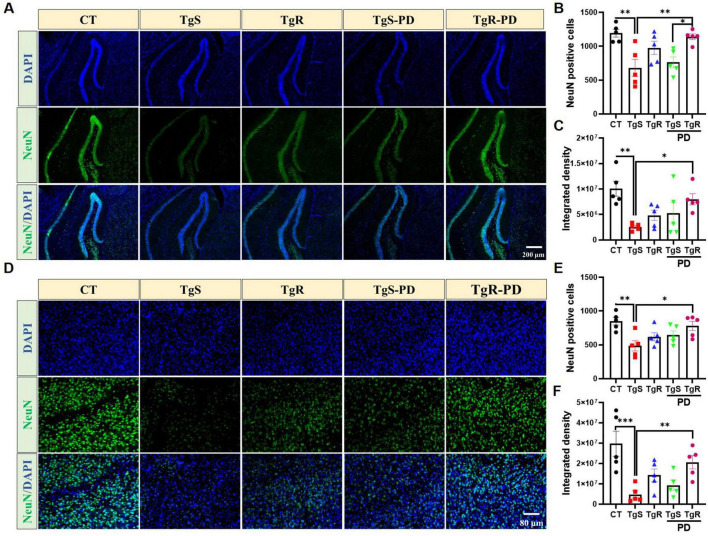
PD and voluntary running protected neurons in the hippocampus and perirhinal cortex. **(A)** Representative NeuN immunostaining images in the hippocampal DG of mice. **(B)** NeuN positive cells in hippocampal DG. **(C)** Integrated density of NeuN in hippocampal DG. **(D)** Representative NeuN immunostaining images in the perirhinal cortex of mice. **(E)** NeuN positive cells in the perirhinal cortex. **(F)** Integrated density of NeuN in the perirhinal cortex. *p < 0.05, **p < 0.01, ***p < 0.001 vs. TgS group. The Kruskal–Wallis test followed by Dunn’s *post-hoc* test was used (mean ± SEM, *n* = 5).

To assess the impact of PD and/or physical running on neuronal apoptosis, we performed immunohistochemical staining with the NeuN antibody. The results revealed that the combined treatment effectively reduced neuronal apoptosis in both the hippocampal DG and perirhinal cortex compared to the TgS group (Figure 6). Specifically, the number of NeuN-positive neurons was significantly higher in the TgR-PD group than in the TgS-PD group (Figures 6A, B). In conclusion, the combined intervention of PD and voluntary running appears to induce 5-HT and DA upregulation and offers neuronal protection, likely contributing to improvements in memory function.

## 4 Discussion

In the present study, we examined the effects of PD nutritional intervention and physical exercise on memory deficits in 5 × FAD mice, an APP/PS1 transgenic model of AD. We found that the combination of PD and voluntary running dramatically inhibited hyperactivation of glial cells, shifted microglia and astrocytes toward beneficial phenotypes, alleviated systemic inflammatory cytokines, promoted Aβ plaque clearance, and restored hippocampal neurotransmitters 5-HT and DA, which ultimately attenuated memory deficits in the 5 × FAD mice. To our knowledge, this is the first study to demonstrate the impact of PD nutritional intervention and physical exercise on learning and memory in AD.

Report has indicated that PD inhibits Aβ-induced oxidative stress and inflammatory response in BV2 cells via activating the Nrf2/HO-1 pathway and suppressing the TLR4/NF-κB signaling pathway (Zhang et al., 2021). In addition, PD ameliorates memory impairment induced by AlCl_3_ and D-galactose via AMPK activation, which mediates the suppression of mitochondrial ROS, inhibition of neuroinflammation, and reduction of neuronal apoptosis (Zhang et al., 2023). However, whether PD modulates glial polarization and neurotransmitter levels in 5 × FAD mice remain elusive. A network meta-analysis noted that short bursts of aerobic exercise improved cognitive performance more effectively than donanemab, lecanemab, aducanumab, or placebo in AD patients, with better tolerability and acceptability (Terao and Kodama, 2024). Although the cognitive benefits of physical exercise in AD model animals are well documented, the involved signaling pathways such as CNS neurogenesis (Norevik et al., 2024), cerebral neuroinflammation modulation (Zhao, 2024), and non-amyloidogenic AβPP processing (Elsworthy et al., 2022), few studies have examined the combination of exercise and medication. For example, swimming combined with clove oil treatment restored Aβ1-42-induced memory deficits by increasing α7nAChR and decreasing NLRP1 and dark cells (Karaji et al., 2023). Similarly, combining luteolin with wheel exercise reduced Aβ levels, glial cell activation, and autophagy in Aβ1-42-induced AD model rats, which was superior to monotherapy (Tao et al., 2023). Moreover, combined aerobic exercise and crocin treatment yielded a more profound effect on cognitive performance, nerve growth factor expression, and tau gene expression in trimethyltin-induced AD rats (Moghadasi et al., 2024). Despite these findings, the impact of PD combined with voluntary exercise on AD remains unexplored. Thus, this study was designed to explore the impact of combined PD supplementation and physical exercise on cognitive ability in 5 × FAD mice. The results highlighted that the synergistic interventions hold great potential to improve memory function.

Research has shown that levels of Aβ40 and Aβ42 were robustly increased from 4 to 8 months in 5 × FAD mice, correlating with elevated reactive GFAP^+^ astrocytes and neurotoxic Iba1^+^ microglia (Forner et al., 2021). Mass spectrometry and bioinformatics analyses demonstrate that the microglial inflammatory response occurs prior to Aβ accumulation (Boza-Serrano et al., 2018). In addition, activated microglia exacerbate AD by triggering reactive astrocytes through the secretion of Il-1α, TNFα, and C1q (Liddelow et al., 2017). Six months old 5 × FAD mice exhibit a significant increase in brain Aβ plaques, an upsurge in neuroinflammatory cytokines, and cognitive decline in behavioral experiments (Girard et al., 2013). Thus, we utilized 5-month-old 5 × FAD mice and comprehensively assessed cognitive changes after a 1.5-month synergistic intervention. A toxicity test revealed that a single oral dose of 2,000 mg/kg PD had no obvious effect on organ weights or histopathological changes (Lee et al., 2011). Our pre-experiments have demonstrated that 5 and 15 mg/kg (p.o.) PD treatment for 2 weeks significantly improved memory deficits in 6–8 months old 5 × FAD mice. Intriguingly, PD treatment slightly reduced body weight, though not significantly, aligning with reports that PD exerts anti-obesity effects in *db/db* mice (Kim et al., 2019). Mild exercise combined with PD treatment markedly decreased body weight, opening up a potential new strategy for anti-obesity. In the open field and water maze tests, a reduction in locomotor activity was observed following PD treatment, likely due to its sedative effects. Previous study has demonstrated that oral administration of platycodon crude saponin effectively prolongs sleep duration in pentobarbital sodium-injected mice (Choi et al., 2004), and PD may be one of the active components contributing to this effect. Additionally, voluntary exercise appeared to counteract the sedative effects of PD.

Despite the fact that clinical trials targeting Aβ have largely been unsuccessful, Aβ plaque aggregation in the brains of AD patients remains one of the key pathological hallmarks of AD (Nehra et al., 2024). Aβ interacts with and activates dynamin related protein 1 (Drp1), which is expressed in the mitochondrial membrane and is important for normal mitochondrial division. This interaction induces mitochondrial dysfunction and synaptic loss (Qi et al., 2019). In addition, Aβ triggers oxidative stress through a variety of pathways, including the activation of nicotinamide adenine dinucleotide phosphate oxidase (Nox) and the production of reactive oxygen species (ROS). This leads to an influx of Ca^2+^, resulting in neuronal injury, apoptosis, or necrosis (Oguchi et al., 2017). Phagocytosis of Aβ by microglia activates the NLRP3 inflammasome and caspase-1, causing the release of IL-1β and inflammatory responses, which further deteriorate AD pathology. Hyperactivated astrocytes, triggered by microglia, produce a large number of pro-inflammatory cytokines such as IL-1β and TNF-α, which suppress astroglial autophagy and block the clearance of Aβ in the brain (Gui et al., 2020). The deposition rate of Aβ1-42 was significantly accelerated in 5 × FAD mice, and reducing Aβ1-42 levels in the brain or serum of AD model rats is highly correlated with cognitive improvement (Oakley et al., 2006). The current study demonstrated that combining PD with physical running dramatically reduced Aβ plaque load in both the hippocampus and cortex of 5 × FAD mice, particularly decreasing the number of small-diameter plaques. Importantly, the TgR-PD group was more efficacious in eliminating 20–40 μm-diameter plaques compared to the TgS-PD group, as well as reducing the total amount of cortical Aβ1-42.

A large number of reactive microglia and astrocytes are observed around Aβ plaques in AD patients, indicating a strong association between Aβ deposition and neuroinflammation (Fakhoury, 2018). Under normal conditions, resting microglia are responsible for removing neuronal debris or remnants via phagocytosis, regulating neuronal homeostasis, maintaining synaptogenesis, and secreting neurotrophic factors (Cserép et al., 2020). Astrocytes, on the other hand, are involved in blood-brain barrier maintenance, synaptogenesis regulation, and neuronal activation (Sofroniew, 2015). Neuroinflammation disrupts these essential functions. Our results indicate that physical exercise combined with PD treatment can inhibit the hyperactivation of astrocytes and microglia in the brains of 5 × FAD mice. Pro-inflammatory cytokines such as TNF-α, IL-1β, and IL-6 were significantly increased, while anti-inflammatory cytokines IL-4 and IL-10 were significantly reduced in AD models (Kaur et al., 2019). ELISA results revealed that physical exercise and PD intervention synergistically decreased serum levels of pro-inflammatory cytokines and increased levels of anti-inflammatory cytokines. Under certain conditions, microglia can differentiate into the M1 phenotype, which secretes high levels of pro-inflammatory cytokines such as IL-6, TNF-α, and IL-1β, or into the M2 phenotype, which produces increased amounts of anti-inflammatory factors like transforming growth factor-β (TGF-β), IL-4, and IL-10 (Liu et al., 2024). Similarly, reactive astrocytes can polarize into either pro-inflammatory phenotype and anti-inflammatory phenotypes. Hyperactivated microglia secrete IL-1α, TNF-α, and C1q, which together induce neurotoxic reactive astrocytes, promoting neuronal death in AD (Liddelow et al., 2017). We demonstrated that physical exercise and PD intervention synergistically promoted the polarization of microglia and astrocytes toward beneficial phenotypes.

Neuronal viability and the neurotransmitter-involved neural networks are crucial for memory formation, consolidation, and retrieval. Aβ-induced neuronal death and disruption of neurotransmitter systems impair neural networks, ultimately leading to memory deficits (Deng et al., 2022). In the present study, we observed a significant reduction in NeuN-positive neurons in 5 × FAD mice. However, combined physical exercise and PD intervention restored hippocampal and cortical neurons. The neurotransmitter 5-HT regulates learning and memory via modulating dopaminergic, cholinergic, and GABAergic signaling. Studies have indicated that 5-HT promotes activation of 5-HT1A receptor, increasing the level of DA in the marmoset brain and enhancing the DA neuronal pathway (Baba et al., 2015). Additionally, activation of the 5-HT4 receptor promotes acetylcholine (Ach) release (Segu et al., 2010). Furthermore, 5-HT activates the 5-HT7 receptor, which increases the release of GABA in the hippocampal CA1 region, enhancing the inhibitory GABA pathway and ultimately improving learning and memory abilities. Investigations have revealed that DA levels are decreased in AD patients, animal models, and those with Parkinson’s disease, resulting in impaired long-term potentiation (LTP) and cognitive decline (Papenberg et al., 2014). DA plays a critical role in regulating neural network activities involved in learning and memory in young animals, but its levels decrease with age. We found that 5-HT and DA levels were significantly reduced in 5 × FAD model mice, but were restored by combined physical exercise and PD intervention. An inverted U-shaped relationship exists between DA levels and synaptogenesis. DA activates the cAMP-PKA pathway via binding to D1/D5 receptors, which promotes the phosphorylation of NMDA and AMPA receptors, improving synaptic plasticity and LTP (Flores-Barrera et al., 2014), and on the other hand, leads to the phosphorylation of DA and cAMP-regulated phosphoprotein (DARPP-32), inhibiting protein phosphatase 1 (PP-1) and activating CREB to induce LTP, thereby enhancing learning and memory functions (Srivastava et al., 2018). However, excessive DA can overactivate D1 receptors, inhibit NMDA receptors, reduce intracellular Ca^2+^ levels below the threshold required for LTP, and induce long term depression (LTD), thereby impairing learning and memory functions (Thirugnanasambandam et al., 2011). DOPAC levels in TgR-PD treated 5 × FAD mice showed an increased tendency compared to the TgS group, suggesting that regulating DA metabolic homeostasis may be a potential strategy for restoring memory ability.

The current study has several limitations. Firstly, as group housing did not permit predictions about the running wheel use of individual animals, and to evaluate whether physical activity alone has beneficial effects, each mouse from each group was singly caged in standard polypropylene cages. Report has shown that single housing exacerbates cognitive impairment by increasing Aβ and calpain activity (Hsiao et al., 2018). While, other studies have demonstrated that long-term voluntary exercise can ameliorate cognitive impairment in singly housed AD mice, future research should aim to minimize the stress associated with prolonged isolation (Belaya et al., 2020; Zhang et al., 2022). As suggested by previous studies, mice could be housed in cages with running wheels for 3 h daily and returned to their original cages for the remaining 21 h (Choi et al., 2018). Secondly, the limited number of mice used in the current study limits its conclusions. Although additional pre-experiments were conducted to confirm the effects of PD or voluntary running on AD using 5 × FAD mice, larger scale, validated studies are needed to improve statistical power and result reliability. Furthermore, questions remain about the direct targets of PD, the role of physical running mediated peripheral-CNS interactions in the amelioration of AD pathologies, and why the combination of PD and voluntary running is superior to monotherapy? Addressing such issues will be an essential task for future research.

Collectively, the present study demonstrated that the combination of PD and voluntary running attenuated cognitive deficits in 5 × FAD mice that is superior to monotherapy. However, our investigation is currently limited in exploring changes in pathological features, and further studies are needed to elucidate the underlying molecular mechanisms. In addition, further clinical and pre-clinical research is required to confirm the efficacy of PD and physical running in AD. Our results highlight that nutritional and physical exercise synergistic interventions hold great potential to treat AD.

## Data Availability

The original contributions presented in this study are included in the article/Supplementary material, further inquiries can be directed to the corresponding authors.
