# Supplementary material for: Platycodin D and voluntary running synergistically ameliorate memory deficits in 5 × FAD mice via mediating neuromodulation and neuroinflammation
Source: Front Aging Neurosci. 2024 Sep 25;16:1451766. doi: 10.3389/fnagi.2024.1451766 (PMC11461226; doi:10.3389/fnagi.2024.1451766)
Supplement: Supplementary file 1 [file Table_1.DOCX]

**SUPPLEMENTARY INFORMATION**

**Platycodin D and voluntary running synergistically ameliorate memory deficits in 5×FAD mice via mediating neuromodulation and neuroinflammation**

Junxin Liu^a^, Jiahui Jiang^a^, Chuantong He^a^, Longjian Zhou^a^, Yi Zhang^a^, Shuai Zhao^a,^*, Zhiyou Yang^a,b^*

^a^College of Food Science and Technology, Guangdong Ocean University, Guangdong Provincial Key Laboratory of Aquatic Product Processing and Safety, Guangdong Province Engineering Laboratory for Marine Biological Products, Zhanjiang Municipal Key laboratory of Marine Drugs and Nutrition for Brain Health, Zhanjiang 524088, China.

^b^Collaborative Innovation Center of Seafood Deep Processing, Dalian Polytechnic University, Dalian 116034, China

*Corresponding author:

Shuai Zhao, PhD, College of Food Science and Technology, Guangdong Ocean University, Zhanjiang 524088, China. E-mail address: zhao_shuai@gdou.edu.cn

Zhiyou Yang, PhD, College of Food Science and Technology, Guangdong Ocean University, Zhanjiang 524088, China. Tel./fax: +86-0759-2396046. E-mail address: zyyang@gdou.edu.cn


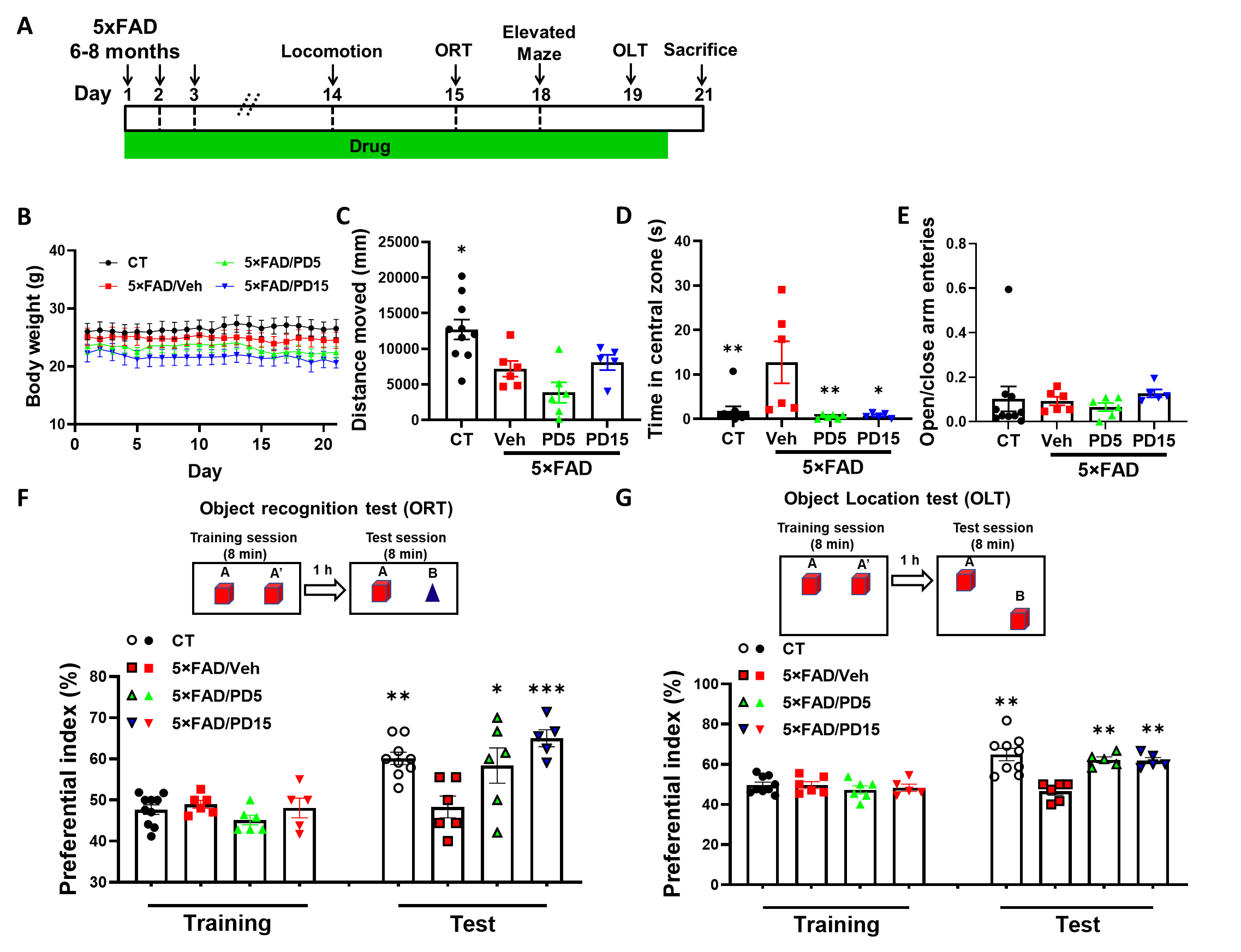


**Fig.1.** The effects of PD on learning and memory function in 5×FAD mice. PD was orally administered to 5×FAD mice (6-8 months old, half male and female) at 5 and 15 mg/kg for 20 days. The locomotion test, object recognition test (ORT), elevated maze test, and object location test (OLT) were performed at day 14, 15, 18, and 19, respectively. (A) The experimental schedule. (B) The body weights during PD treatment. (C) Distance moved in the open field test. (D) Time in central zone in the open field test. (E) The ratio of open and close arm entries in the elevated maze test. (F) The novel object recognition test. (G) The object location test. *p < 0.05, **p < 0.01, ***p < 0.001 vs 5×FAD/Veh group. The Kruskal-Wallis test followed by Dunn’s post hoc test was used for one-factor designs, and the Scheirer-Ray-Hare test (an extension of the Kruskal-Wallis test) followed by Dunn’s post hoc test was used for experiments with two-way designs (mean ± SEM, n = 5-10).


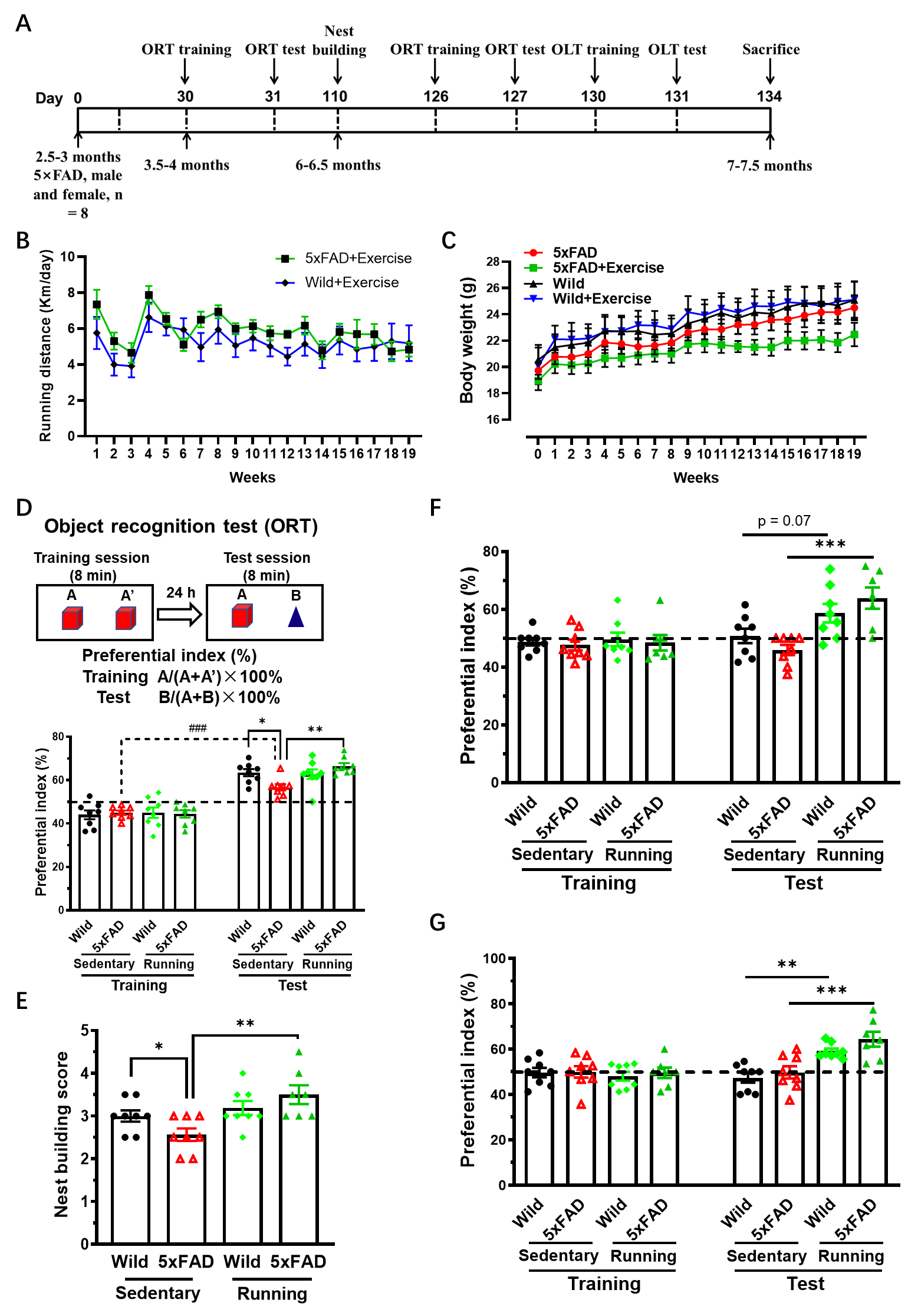


**Fig.2.** Voluntary running attenuated memory deficits in 5×FAD mice. Experiments were performed using 5×FAD mice (2.5-3 months old, half male and female, n = 8) and littermate wild-type C57BL/6 mice (2.5-3 months old, half male and female, n = 8). They were randomly divided into 4 groups including sedentary 5×FAD group, sedentary wild-type group, voluntary running 5×FAD group, and voluntary running wild-type group. (A) The experimental schedule. (B) The running distance. (C) The body weights during experiment. (D) The novel object recognition test on day 30. (E) The nest building test on day 110. (F) The novel object recognition test on day 126. (G) The object location memory test on day 130. *p < 0.05, **p < 0.01, ***p < 0.001 vs 5×FAD group. Two-way ANOVA *post-hoc* Šidák’s multiple comparisons test (mean ± SEM, n = 8).

**Fig.3.** PD and voluntary running decreased the body weights in 5×FAD mice. *p < 0.05, **p < 0.01, ***p < 0.001 vs TgS group. Two-way ANOVA *post-hoc* Fisher’s LSD test (mean ± SEM, n = 8).
